# Increased risk of ischemic stroke in patients with burn injury: a nationwide cohort study in Taiwan

**DOI:** 10.1186/cc14550

**Published:** 2015-03-16

**Authors:** TY Hung, YC Su

**Affiliations:** 1 Zhongxing Branch of Taipei City Hospital, Taipei, Taiwan; 2 Dalin Tzu Chi Hospital, Buddhist Tzu Chi Medical Foundation, Chiayi, Taiwan

## Introduction

The results of studies attempting to assess the risks of ischemic stroke in patients with burn injury have been conflicting. We investigated the risks of ischemic stroke in hospitalized burn injury patients in Taiwan to evaluate whether the risk is higher compared with the general population.

## Methods

The data from 1 million National Health Insurance bene ficiaries were utilized. All adult beneficiaries were followed from 1 January 2005 until 31 December 2012 to identify those who developed ischemic stroke. Meanwhile, each identified patient with burn injury was matched with 100 unexposed patients based on the high-dimensional propensity score. Cox regression models were applied to compare the hazards of ischemic stroke in the matched cohorts.

## Results

**Figure 1:**
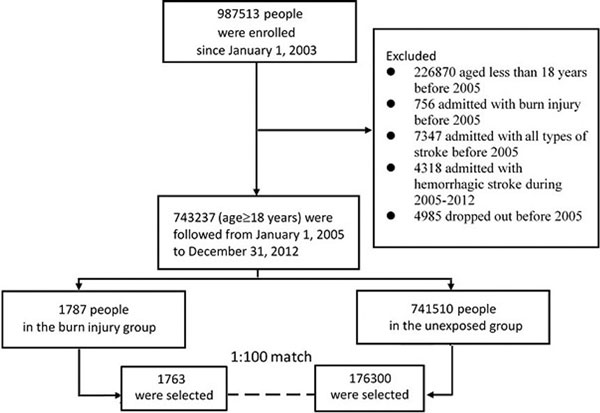
**Patient flowchart of enrollment**.

**Figure 2:**
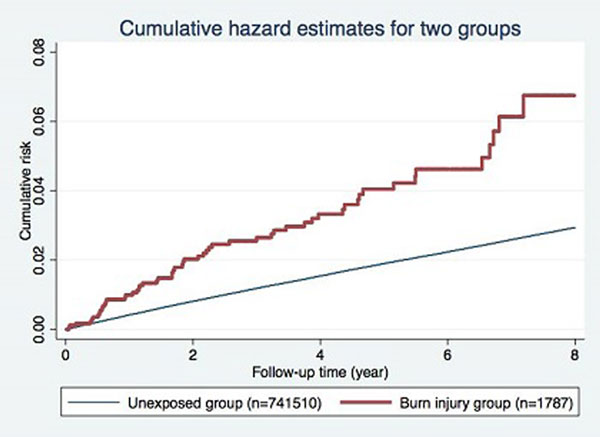
**Burn injury patients have higher adjusted hazard ratio of ischemic stroke**.

A total of 743,237 patients were enrolled. After matching, 1,763 burn injury patients and 176,300 unexposed patients were selected. The adjusted hazard ratio of ischemic stroke was significantly increased in burn injury patients (1.84; 95% CI, 1.43 to 2.36). Such phenomenon remained significantly after 12 months (1.54; 95% CI, 1.11 to 2.13). See Figures 1 and 2.

## Conclusion

The risk of ischemic stroke is higher in patients hospitalized with burn injury than in the general population, and the effects may be extended longer than expected previously.
